# Spatial distribution, prevalence and potential risk factors of Tungiasis in Vihiga County, Kenya

**DOI:** 10.1371/journal.pntd.0007244

**Published:** 2019-03-12

**Authors:** Ruth Monyenye Nyangacha, David Odongo, Florence Oyieke, Christine Bii, Erastus Muniu, Stanley Chasia, Missiani Ochwoto

**Affiliations:** 1 Center for Traditional Medicine and Drug Research, Kenya Medical Research Institute, Nairobi, Kenya; 2 School of Biological Sciences, University of Nairobi, Nairobi, Kenya; 3 Center for Microbiology Research, Kenya Medical Research Institute, Nairobi, Kenya; 4 Center for Public Health Research, Kenya Medical Research Institute, Nairobi, Kenya; 5 Department of Geosciences and Environment, Technical University of Kenya, Nairobi, Kenya; 6 Production Department, Kenya Medical Research Institute, Nairobi, Kenya; University of California San Diego School of Medicine, UNITED STATES

## Abstract

**Background:**

Tungiasis is a parasitic disease caused by the sand flea *Tunga penetrans* also known as jigger flea. Communities living in precarious conditions in tropical and sub tropical countries bear the brunt of the infection. The main objective of this study was to determine the burden of Tungiasis in Vihiga County in Kenya.

**Methods:**

This was a cross-sectional study conducted in 21 villages in 3 Sub-locations in Vihiga County, western Kenya. A total of 437 participants, 5 years old and above were clinically examined for the presence of tungiasis after consenting to take part in the study. Diagnosis was made following standard methods. A semi- structured questionnaire was administered to assess socio-demographic factors, housing, presence and ownership of animals, knowledge and practice related to tungiasis. Data were analyzed using bivariate and multivariate regression analysis. GIS was used to map the geographic distribution of tungiasis in the area.

**Results:**

The overall prevalence was found to be **(21.5%; 95% CI: 17.7–25.3%)**. The cases were analysed and visualized in a map form. Multivariate analysis suggested that the occurrence of tungiasis was associated with variables that indicated low economic status (like a monthly income of Ksh ≤ 1000 (adjusted odds ratio 27.85; 95% CI: 4.13–187.59), earthen floor (0.36; 0.13–1.024) and lack of toilet facilities (4.27; 0.82–22.34), age of participant ≤14 (27.414; 10.02–74.99), no regular use of closed footwear (1.98; 0.987–3.97) and common resting place inside the house (1.93; 0.96–3.89).

**Conclusions:**

Tungiasis is an important health problem in Vihiga County occasioned by the low economic status of the people affected. Factors that point to poverty contribute to the occurrence of tungiasis. These findings suggest a need to design control strategies for tungiasis that are cost effective and easily accessible.

## Author summary

Tungiasis even though curable, continues to cause a lot of morbidity and in some cases death to millions of people living in tropical and subtropical countries. The global burden of tungiaisis is not known unlike for most diseases which attract a lot of attention and funding. There are also gaps on the exact geographical distribution of this disease and the communities affected, hence it is largely ignored and neglected by the scientific community, the health sector, pharmaceutical industries and policy makers. As a consequence, there are no universally accepted methods to treat and control tungiasis. The study attempted to close this gap by combining geographic information system and questionnaire data to visualize the burden and risk factors of tungiasis in Vihiga County, Kenya. The researchers found that tungiasis is an important health burden in Vihiga County, Kenya and that its occurrence was associated with factors that point to poverty. These findings suggest a need to design control strategies for tungiasis that are cost effective and easily accessible by the communities affected.

## Introduction

Tungiasis also referred to as jigger infestation is a neglected tropical disease due to the fact that it typically occurs in populations living in poverty, with poor housing, inadequate sanitation and in close contact with domestic animals akin to other neglected diseases. It is however not recognized officially by the world health organization (WHO) [1]. The disease affects communities living in precarious conditions trapping them in a vicious cycle of poverty and disease [2]. It is endemic in tropical and subtropical countries [3]. Identification of the exact areas affected is still a problem in most countries. The burden of tungiasis in the world is also unknown and has never been assessed [1]. In Kenya the few studies carried out on affected communities have documented a prevalence range of between 19 to 40% [4, 5, 6, 7]. During peak transmission, children and the elderly bear the brunt of the infection. In central Kenya, a prevalence of 57% in children between the ages of 5–12 years old has been recorded [5]. In endemic communities in Brazil and Nigeria prevalence as high as 60% has been reported in children as well [8, 9].

Factors that have been reported to aggravate the problem include earthen and dusty floors, non regular use of closed footwear, common resting place outside the house and pigs on the compound [10]. Sleeping on the floor and crowded sleeping areas have also been cited [7]. Others are living with animals in the same house [11], occurrence of animals like dogs, cats, rats, cows, goats, chicken in the compound [12, 13]and low education level [14].

Information on the spatial distribution of tungiasis is nonexistent save for a recent study which seeked to create web based GIS applications for the disease [15]. When the geographical distribution of neglected diseases is well understood, control measures are greatly improved [16]. For instance, due to limited resources in Sub Saharan Africa, health interventions at the national level in most countries target high transmission areas leaving out low or zero transmission areas [17]. For this to be possible, data on the geographical distribution of neglected diseases and the communities affected is important. Considerable progress on mapping of neglected diseases has been made [18, 19]. However a lot still needs to be done for diseases like tungiaisis so as to understand where the communities affected live and the magnitude of the infection.

For as long as objective and clear data on the geographical distribution and burden of tungiasis remains elusive, the communities affected continue to bear the brunt of the disease in silence. Areas that can be targeted to control or eliminate this ectoparasitosis are gaps that should be closed as well. This study describes the distribution, prevalence and potential risk factors of tungiasis in western Kenya.

## Methods

### Study area and population

This study was conducted in Vihiga County in Western Kenya which has 5 constituencies namely Sabatia, Vihiga, Emuhaya, Luanda and Hamisi. It is one of the most densely populated rural areas in Kenya. It has a population of 554,622 with 45% of these being 14 year olds and below. A large percentage of the population (79.4%) stay in homes with earthen/mud floors [20]. The poverty levels are high (41%) and the GDP per capita income was reported as US $ 1,103 in 2013 [21]. The area has two rainy seasons. Long rainy season in April, May and June and the short rains in September, October and November.

### Study design

**Fig 1:**
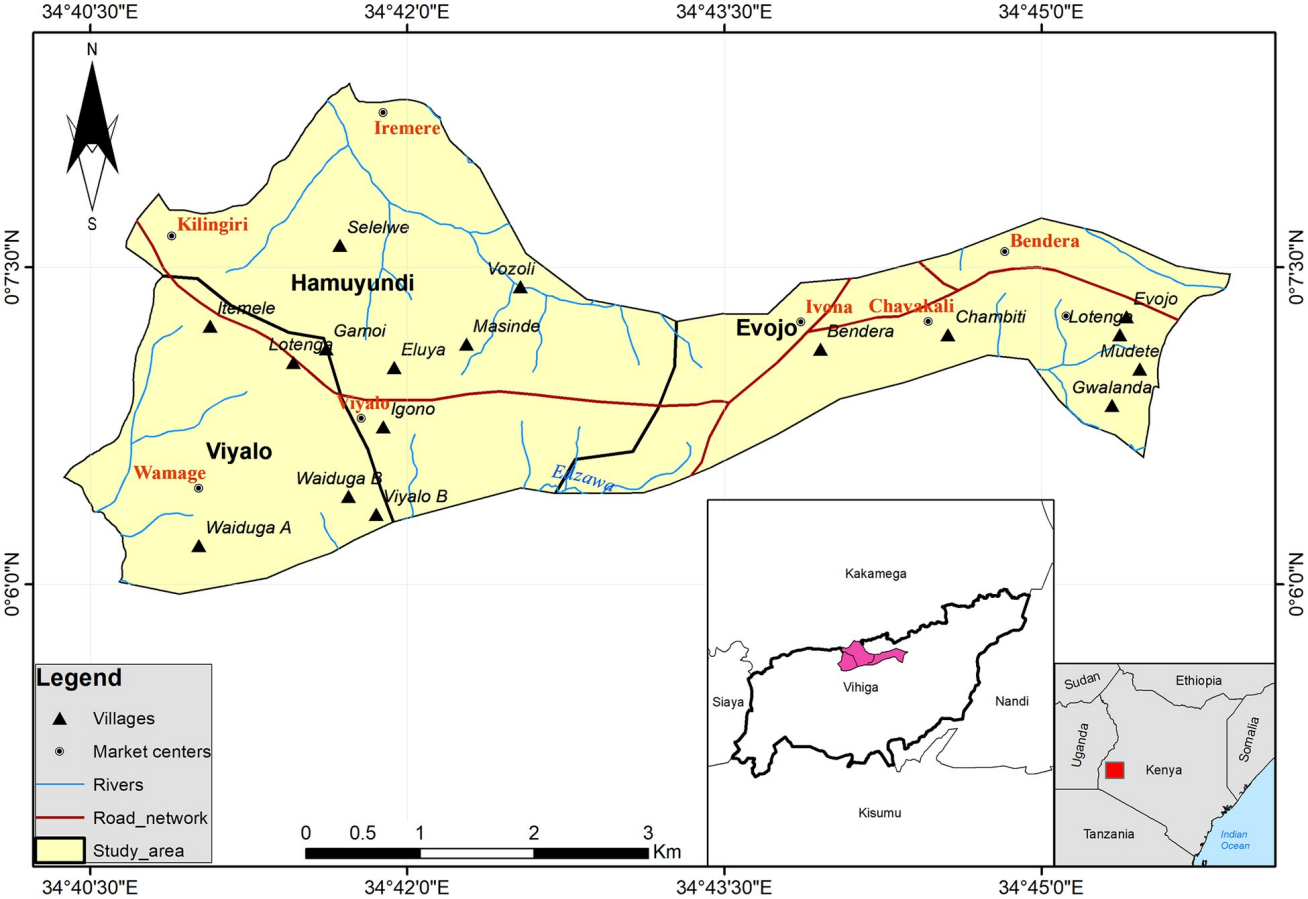
Study area map.

This was a community based, cross- sectional study that took place between February and March 2016, a dry season when prevalence of Tungiasis is known to be at its peak. The study was carried out in 21 villages in 3 sub locations namely, Hamuyundi, Viyalo and Evojo which were randomly selected from Sabatia constituency, Vihiga County, Kenya (Fig 1). A listing of all households in the 3 sub-locations was carried out and after proportionately allocating number required per sub-location, selection was done using systematic sampling method with a random start. In each sampled household, one eligible participant was sampled at random.

The sample size was determined using the Daniel, 1999 formula which uses prevalence as the P value [22]. The prevalence of tungiasis ranges between 15% to 60% and therefore a proportion of 50% was used to draw the sample size [23; 24]. The calculated sample size was 385 plus 20% to account for non-response or missing values [24]. Thus total number of participants was adjusted to 460.

The inclusion criteria for the study population was all residents in the randomly selected households aged 5 years and above, who were willing and who had resided in the County in the last 24 months. Residents below the age of 5 years in the randomly selected households, individuals who declined or their minors to participate in the study, and none residents were excluded from the study.

A total of 437 participants consented and were enrolled in the study. Their hands and feet were clinically examined for the presence of vital, egg-producing, involuting, or dead fleas once consent was obtained. The following findings were diagnostic for tungiasis: an itching red-brownish spot with a diameter of 1–3 mm; a circular lesion presenting as a white patch with a diameter of 4–10 mm with a central black dot; a black crust surrounded by necrotic tissue; and partially or totally removed fleas leaving a crater like sore in the skin [25].

A pre-tested semi-structured questionnaire was administered to the head of the household or the oldest adult in the family, in English, swahili or luhya, the local dialect, upon consenting. This was done by researchers with the help of two trained community health workers. The community health workers were trained for 2 days on the questionnaire, consenting process and what the whole study entailed by the research staff. The information collected consisted of four categories. (1) Socio-demographic factors such as sex, age, education. (2) Housing and associated factors such as type of construction of the house, type of floor inside house, sanitary conditions, presence of electricity and waste disposal. (3) Ownership and presence of domestic animals. (4) Knowledge and practices related to Tungiasis such as knowledge on transmission, regular use of footwear, common resting place, preventive measures and treatment. A household was revisited incase a family member was absent.

### Household mapping

Details about the location of the participant’s homes were mapped using a hand held Garmin eTrex 10 global positioning system (Garmin international inc., Olathe, Kansas). The waypoints data representing household location and elevation information, were collected using the World Geodetic Survey (WGS 1984) datum using the UTM projection system zone 36 north.

### Data analysis

Data was entered into excel database and after cleaning, transferred to SPSS version 21.0 for data analysis. Data analysis involved descriptive statistics (means, standard deviations, medians and frequency distributions, etc.) as well as computation of prevalence rate ratios (PRR). Variables that were associated with tungiasis following bivariate analysis level at p<0.25, were entered into a Binary logistic regression model using backward elimination method and adjusted odds ratios calculated. Multicollinearity among explanatory variables was tested using Spearman rank correlation coefficient (ordinal variables) and chi-square test (nominal variables). All variables that remained in the model are presented.

GIS analysis was carried out using Arc GIS 10.5 from Esri. The basemap, from the topographical map of the area at a scale of 1:50,000, was projected to the Universal Transverse Mercator (UTM) projection system and used to digitize layers i.e. roads, rivers, towns. The GPS coordinates associated with Tungiasis cases were plotted on to the GIS software, projected to UTM system and converted to point shapefiles representing the distribution of cases. The tungiasis case concentration map was produced by interpolating the number of cases recorded per site using the inverse distance weighting (IDW) algorithm tool found in ArcGIS Spatial Analyst tool—a spatial autocorrelation method that interpolates unknown values/cases based on values/cases from nearby known locations [26]. This method assumes the existence of a spatial pattern and similarity for cases occurring at close proximity. A semivariogram was used to assess spatial autocorrelation. The number of cases represented the magnitude in the input point feature layer. The output was a floating point interpolated raster surface in form of a heat map showing the clustering/concentration of the cases per village in the study area.

### Ethics approval and consent to participate

Approval for this study was sought from the Kenya Medical Research Institute (KEMRI) Scientific and Ethics Review Unit (SERU). Approval number KEMRI/SERU/CTMDR/015/3116. Informed written consent was obtained from all participants including children, where the guardian provided an informed consent on their behalf. All data analyzed were coded and identity of participants kept confidential. All participants were treated for tungiasis according to the National Policy Guidelines on Prevention and Control of Jigger Infestations in Kenya [27]. This was by (removing the embedded flea with a sterile needle and disinfection of the skin lesion) or bathing the affected area in 0.05% potassium permanganate for 10 minutes. A nurse who was part of the team also vaccinated the participants against tetanus. Severe cases were referred to the health center by Community Health Extension Workers from the study area.

## Results

A total of 437 people consented and participated in the study with 231 (52.9%) being males and 206 (47.1%) females. Out of the 437 participants, 94 (21.5%; 95% CI: 17.7–25.3%) were infested with jiggers (25.1%; 95% CI: 19.5–30.7% of males compared to 17.5%; 95% CI: 12.3–22.7% of females). The age specific prevalences are shown on Table 1.

**Table 1:** Prevalence of Tungiasis by age group.

Age group in years	Examined N	Positive cases	Prevalence (%)	95% Confidence Interval	
				Lower	Upper
5–9	33	31	93.9	85.7	102.1
10–14	21	15	71.4	52.1	90.7
15–19	8	6	75.0	45.0	105.0
20–39	85	11	12.9	5.8	20.0
40–59	146	11	7.5	3.2	11.8
≥60	144	20	13.9	8.2	19.6
Total	437	94	21.5	17.6	25.4

### Spatial distribution of Tungiasis in the study area

**Fig 2:**
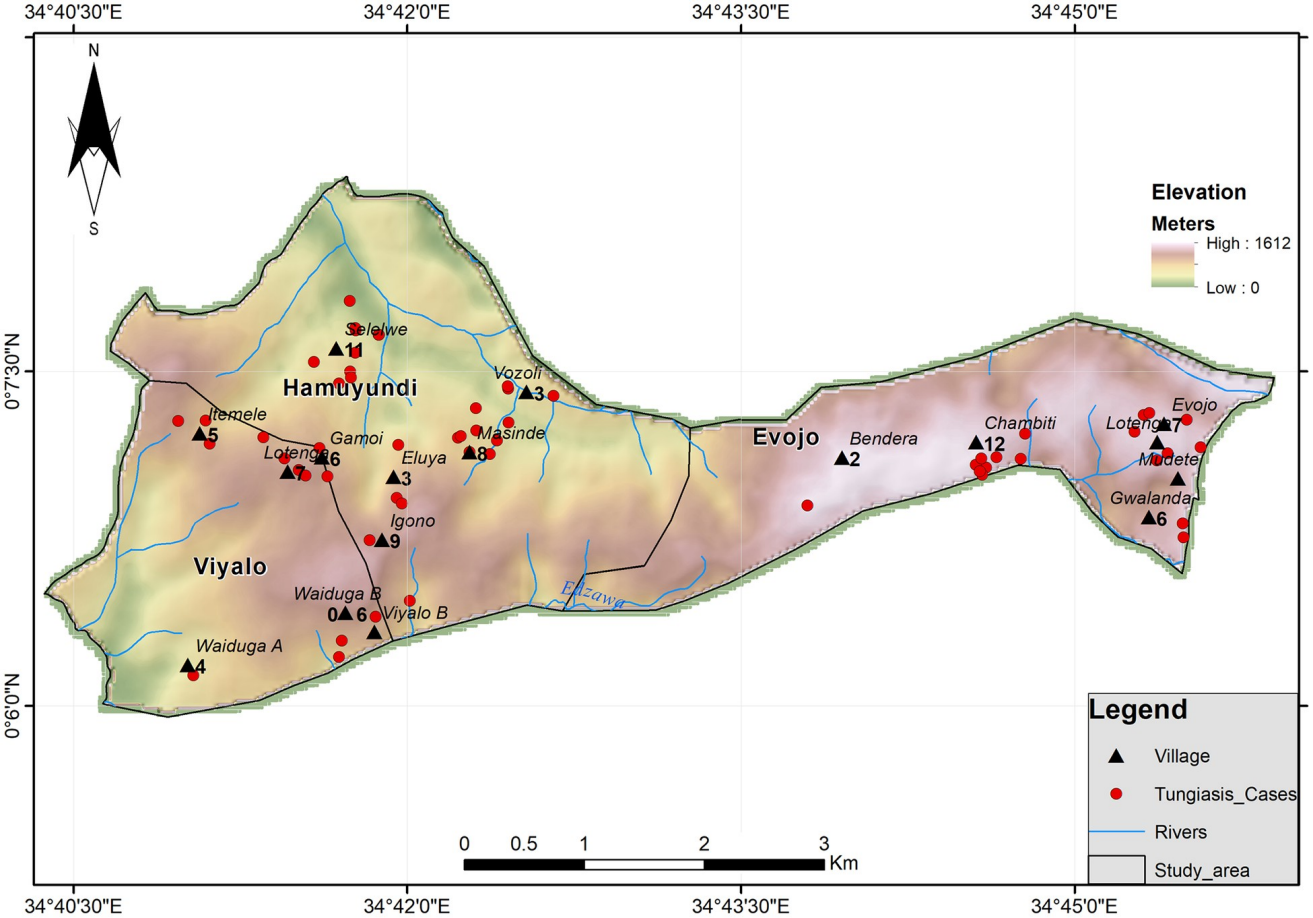
Distribution of cases in relation to elevation.

**Fig 3:**
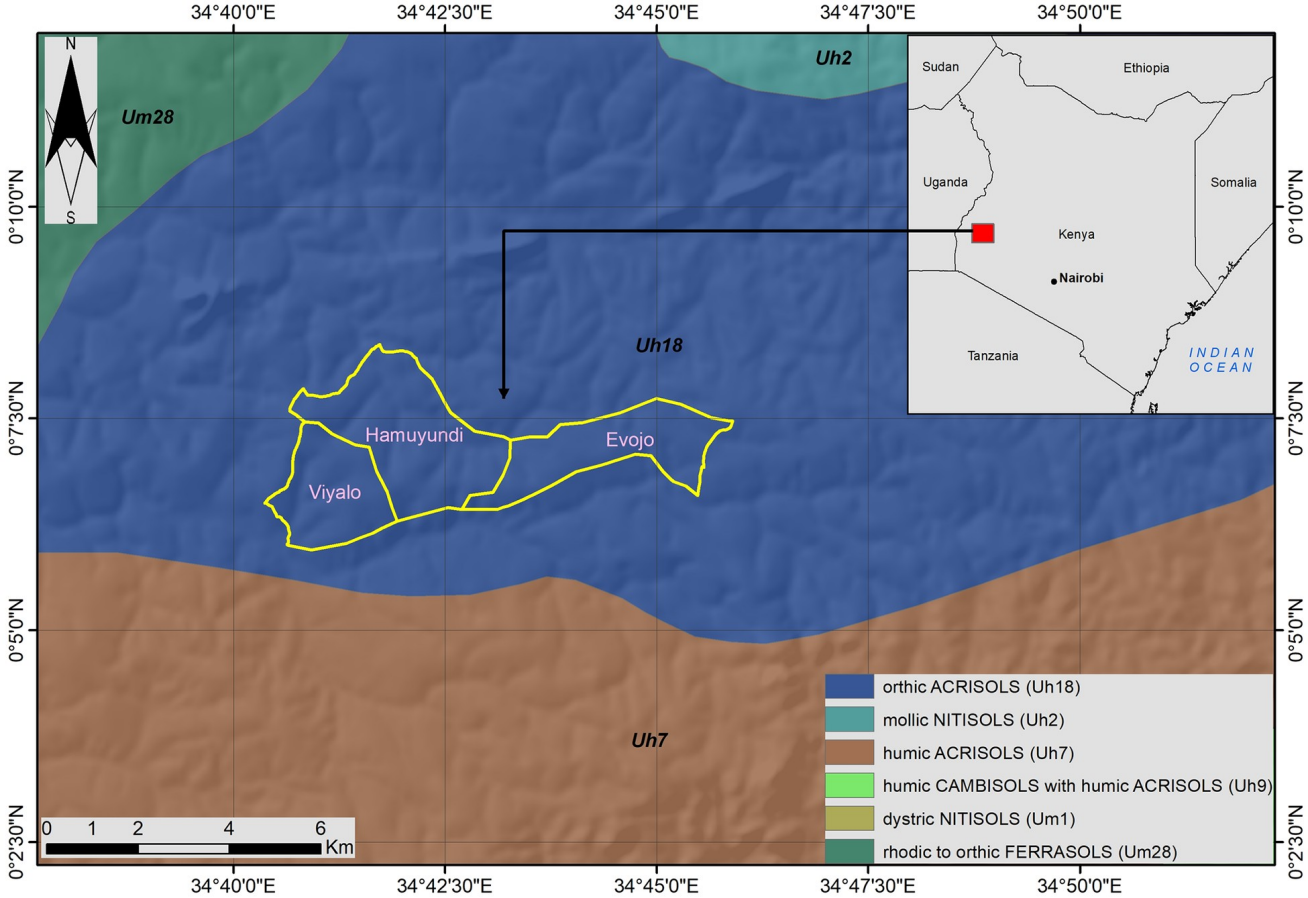
The study area relative to the soils.

Visual investigation of the distribution of cases did not reveal any obvious spatial pattern in terms of the changes in elevation. There is similar distribution in both the low and high altitudes (Fig 2). The spatial dependence of tungiasis cases was further tested using a semi-variogram in ArcGIS (version 10.5) Spatial Analyst tool. A lag size of 0.0074196 was used on the semi-variogram to test autocorrelation in tungiasis concentration. The semivariogram revealed no spatial autocorrelation which could indicate that tungiasis prevalence in the study area might be random. The soil type in the three sub locations did not vary, it was mainly observed to be sandy clay (Fig 3).

**Fig 4:**
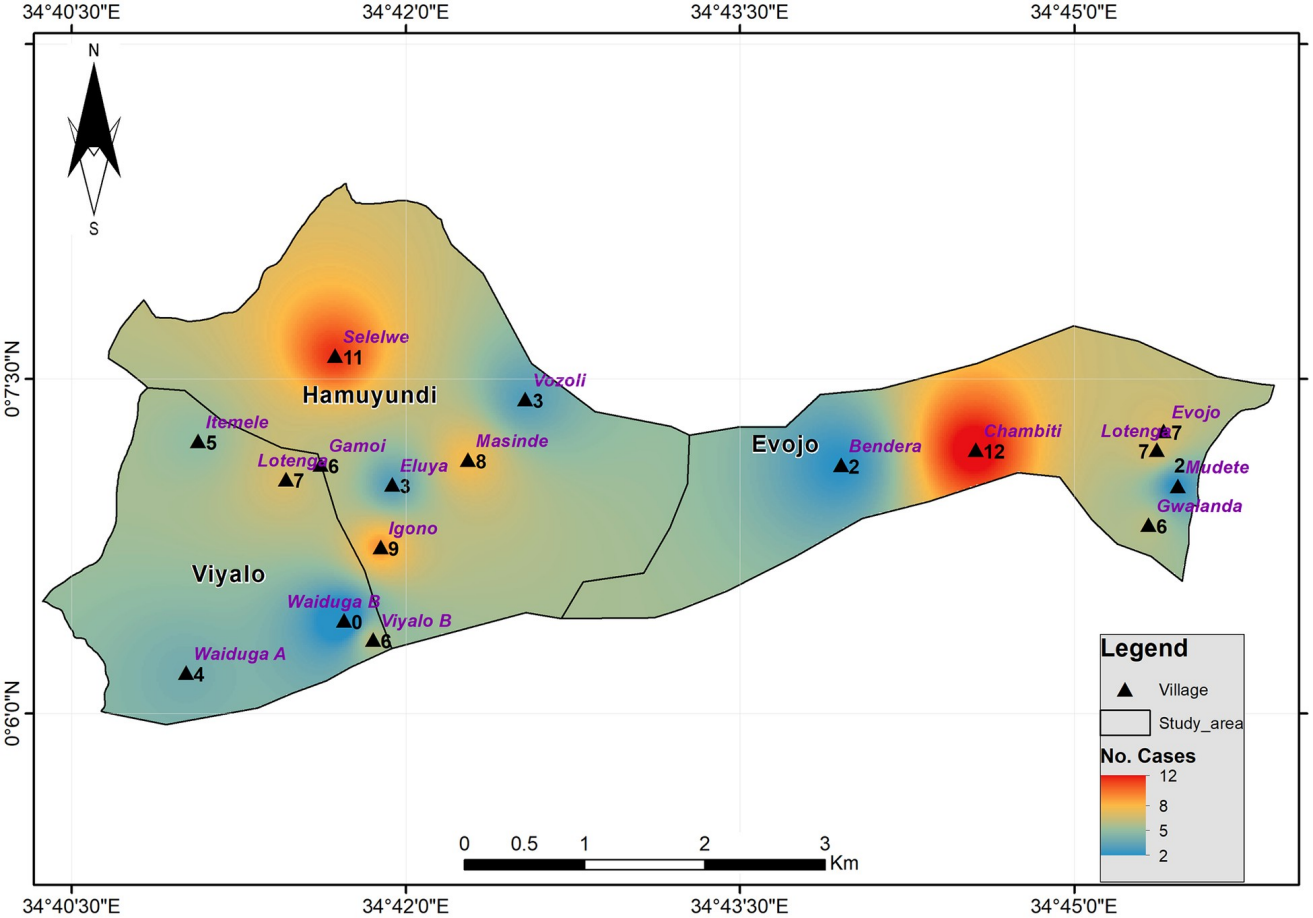
Distribution of cases.

Among the households selected in the three sub-locations, the villages with the highest concentration of cases were Chambiti and Selelwe villages (Fig 4). Five of the villages Wamage, Kivagala B, Walduga B, Kivagala A and Galoni had no jigger infestation.

### Factors associated with tungiasis

In the bivariate analysis, the age group ≤14, school going, self-treatment, occasional or not using closed footwear were shown to be significantly associated with tungiasis (Table 2). Others were being illiterate or low literacy level, bathing without soap, common resting place inside the house, having rats in the compound, lack of electricity and lack of a toilet. When it came to knowledge and practice associated with tungiasis, 45% of the participants interviewed had no knowledge on its transmission.

**Table 2:** Bivariate analysis of factors associated with *tungiasis*.

Factor		Total examined	% Positive	P-Value	Prevalence Rate Ratio
**Socio-demographic factors**					
Gender	Male	231	25.11	0.053	1.44
	Female	206	17.48	Reference	
Age group (Years)	≤14	54	85.19	<0.001	4.66
	15–39	93	18.28	Reference	
	≥40	290	10.69	<0.001	0.58
Education	Primary school completed	227	17.62	Reference	
	Illiterate/primary school not completed	210	25.71	0.040	1.46
Occupation	School going	58	84.48	<0.001	7.89
	Worker/employed	28	10.71	Reference	
	Unemployed/Retiree	148	9.46	<0.001	0.88
Income (Ksh/month)^a^	<1000	255	20.78	0.66	2.03
	1000–5000	141	25.53	0.16	2.49
	>5000	39	10.26	Reference	
Religion	Muslim	5	0.0	0.24	0
	Christian	424	21.23	Reference	
	Other	8	50.0	0.048	2.36
**Housing and associated factors**					
Type of floor	Earthen	390	22.31	0.243	1.49
	Cemented	47	14.89	Reference	
Type of house	Permanent	42	14.29	Reference	
	Semi-permanent	395	22.28	0.231	1.56
Toilet	Pit latrine/wc	425	20.71	Reference	
	None	12	50.0	0.015	2.41
Water supply	Exclusively river	154	25.97	0.094	1.04
	Well and river	132	15.91	0.06	0.72
	Exclusively well/tank	150	22.0	Reference	
Distance from river	<15min	165	20.0	0.55	0.89
	≥15min	272	22.43	Reference	
Waste disposal	Burnt	89	21.35	Reference	
	on compound	256	24.61	0.06	1.15
	outside compound	82	14.63	0.09	0.69
Electricity	No	388	23.19	0.016	2.84
	Yes	49	8.16	Reference	
**Animals on compound**					
Pigs	Yes	20	35.0	0.133	1.68
	No	417	20.86	Reference	
Dogs	Yes	99	20.20	0.719	0.92
	No	338	21.89	Reference	
Cats	Yes	139	16.55	0.053	0.69
	No	298	23.83	Reference	
Rats	Yes	165	31.52	<0.001	2.04
	No	272	15.44	Reference	
Cows	Yes	256	19.14	0.14	0.77
	No	180	25.0	Reference	
Goats	Yes	79	15.19	0.131	0.66
	No	358	22.91	Reference	
Chicken	Yes	127	18.89	0.40	0.84
	No	310	22.58	Reference	
**Knowledge and practice**					
Knowledge on what would cause *tungiasis*	Soil/Animals	241	22.82	Reference	
	Other/Don’t know	196	19.89	0.46	0.87
Type of treatment	Hospital personnel	9	55.56	Reference	
	Self-treatment	248	32.26	<0.001	0.58
	None (no response)	180	5.0	<0.001	0.09
Prevention used	Commercial insecticides	348	19.83	Reference	
	Water/other/none	89	28.09	0.09	1.42
Use of closed footwear	Regularly	163	13.50	Reference	
	No/occasionally	274	26.28	0.002	1.95
Resting place	Inside the house	108	28.70	0.036	1.49
	Outside the house	329	19.15	Reference	
Bathing with soap	No	5	80.0	0.008*	3.84
	Yes	432	20.83	Reference	

*Fisher’s Exact Probability

^a^Ksh 100 is equivalent to ~ 1 USD

In the multivariate analysis, the occurrence of tungiasis was significantly associated with variables that indicated low economic status (like monthly income <1000 adjusted OR 27.85 (4.13–187.59) and age (≤14 years) of participant adjusted OR 27.414 (10.02–74.99) (Table 3). Others retained in the model though not significant were earthen floor, toilet availability, occasional or not using closed footwear and common resting place inside the house.

**Table 3:** Multivariate analysis of potential risk factors of tungiasis.

Variable	Categories	Β	S.E. (β)	p-value	AOR	95% CI for AOR	
						Lower	Upper
Age	<14	3.31	0.51	< 0.0001	27.414	10.02	74.99
	15–39	Reference					
	40 & above	-0.53	0.36	0.141	0.590	0.292	1.192
Income	<1000	3.33	0.97	0.001	27.850	4.13	187.59
	1000–5000	1.40	0.73	0.056	4.054	.964	17.05
	>5000	Reference					
Using closed footwear	No	0.683	0.36	0.054	1.98	.987	3.97
	Yes	Reference					
Type of floor	Earthen	-1.019	0.53	0.055	0.36	0.13	1.024
	Cemented	Reference					
Toilet	None	1.452	0.84	0.085	4.27	0.82	22.34
	Pit latrine/WC	Reference					
Common resting place	Inside house	0.66	0.36	0.064	1.93	0.96	3.89
	Outside house	Reference					

β = regression coefficient; S.E. = Standard error of β; AOR = Adjusted Odds Ratio; CI = Confidence Interval

## Discussion

This study found a prevalence of tungiasis at 21.5% among the study participants. This was similar to what was reported in an earlier community based study in Kenya, which had a prevalence of 25% [7]. It was however lower than what was reported in other countries like Nigeria 45.2% [9] and Tanzania 42.5% [28].

Using GIS, cases were analyzed and plotted in a map form. It was easy to visualize and quickly identify areas that would need to be prioritized when carrying out intervention measures in the area thereby maximizing treatment and control outcomes in the face of limited resources. Out of 21 villages, 5 recorded zero cases of tungiasis. It will be interesting to know what this could be attributed to since from the soil map the soil type was similar to areas that had cases. The houses were also very much the same. Heteregenous distribution of tungiasis in endemic communities has previously been reported [7].

Animals are known to be reservoirs for tungiaisis [29]. In this study presence of cows, pigs, dogs, goats, cats, chicken and rats in the compound were assessed as potential risk factors for tungiasis infestation. In the bivariate analysis only rats were found to be significant in this setting. This corroborated the finding of Heukelbach et al., in Brazil which found that slum rats had a *Tunga penetrans* infestation of 41.2% [29]. In other settings various animals have also been implicated for instance pigs [30] in Nigeria, Dogs [31] in Brazil and goats [32] in Uganda.

Other variables that were seen to be significant in the bivariate analysis were the age group ≤14, school going, occasional or not using closed footwear, being illiterate or having not completed primary school, bathing without soap, having a common resting place inside the house, lack of electricity and lack of a toilet. The significance of the age group ≤14 and school going variables in this study is consistent with a previous study [5] which reported a high prevalence of tungiasis in children of this age group. In addition, the prevalence of 5–14 year olds in the current study was found to be approximately 5 times higher than the prevalence in 15–39 year olds. This can be attributed to the fact that they are more exposed due to them playing barefoot [10] and also getting reinfected at school in endemic areas.

Bathing without soap is a factor that has been elucidated in previous studies as well [7, 13]. Questions abound as to if the action of washing up in a proper way may contribute in a way to the reduction of the occurrence or intensity of tungiasis infestation in humans. Are there aspects that this simple action could contribute to reducing infestation rates in endemic communities? This is a gap that should be investigated further and if found to be plausible, integrated in the control programs of this infestation. Good hygiene practices have been shown to reduce the incidence of several diseases including eye and skin infections like trachoma and scabies [33].

Having a common resting place inside the house was significant in this study. It has long been suspected that transmission of tungiasis could happen indoors (10). This is in line with a study done in Brazil [34] where living stages of the flea were present in soil samples collected indoors and absent in soil samples collected outdoors. In the current study, it was observed that the residents preferred staying indoors even during the day when it was very hot. The compounds are very small given that the area is densely populated. Consequently there are fewer shade areas to rest outside during the day. In Kenya most outreach groups or campaigns to assist the affected communities only focus on the treatment of the participants given that there are still no standard regulations on the treatment, control or prevention of the infestation [7]. Intervention strategies would be greatly enhanced if spraying or dusting of houses with insecticides is integrated in mass control programs. In contrast, a study done in Nigeria [10], found that having a common resting place outside the house was greatly associated with tungiasis. Taken together, it seems plausible that the common feature here is that transmission takes place where patients spend most of their time since the eggs falling onto the ground complete their lifecycle and go back to the human host. If this is true then schools should be looked at as possible venues of transmission and investigated further in longitudinal studies.

The other factors (lack of a toilet, lack of electricity and being illiterate or having not completed primary school) pointed to low economic status. This upheld a previous study [13].

In the multivariate analysis only the age and the monthly income bracket of <1000 remained significant. The type of floor, not using closed footwear regularly, lack of a toilet and having a common resting place inside the house were retained in the model indicating they had a contribution though not significant. They have been shown to be significant in other studies [10, 13] hence can be investigated further in longitudinal studies. Variables retained in the model apart from age are indicative of low economic status. This corroborates the finding of [5, 10]. Tungiasis is certainly a poverty related disease [12].

Almost a half, 45% of the participants interviewed had no knowledge on the transmission of tungiasis. Some of the answers that were given were that it’s brought about by witchcraft, it is inherited or it is a curse. Some especially the elderly reported that infestation with the flea is usually a sign that they are almost dying and so they were awaiting their fate. This is consistent with a study in central Kenya that sort to shed light on stereotypes surrounding tungiasis in Kenya [35]. In eastern Uganda as well a study reported 19.9% of the participants perceived tungiasis as a curse [36]. It was observed in the current study that the people affected suffered a lot of stigmatization and children had dropped out of school due to this. The community should be educated on the fact that this is a parasitic disease that can be treated. The strength of controlling this infection just like in other diseases lies in demystifying it and ensuring that the communities affected understand its transmission dynamics. This will consequently improve the treatment and control outcomes.

The study had a few limitations. Children under 5 years were not included in this study. They were however observed to be affected by tungiasis and should be included in community based studies. Being a cross sectional study the aspect of cause and effect could not be investigated. This is because cross sectional studies are only able to establish an association or a relationship between two or more variables. For a study to claim causality which means that changes in one variable measured directly caused changes in another, a longitudinal study has to be carried out. Future studies can attempt to fill this gap by conducting longitudinal studies on variables of interest. The study did not spatially represent the risk factors because the study area was not big enough to see distinct variability in the data sets for instance topography and soil type among others in relation to tungiasis. The study area was approximately 19.81Km^2^. For this reason, in a small to moderate scale, the risk factors identified by the bivariate and multivariate analysis might represent more useful predictors for control and intervention than when spatially modelled [37]. It was also beyond the scope of this study to develop weights and indices for the different variables which would enable the study to model spatially the risk factors associated with tungiasis distribution in the area. Nevertheless the study was able to use a descriptive interpolation technique using the inverse distance weighting (IDW) method to represent the occurrence of tungiasis in the area in form of a heat map, which may serve to generate interest in the use of GIS in studying tungiasis. The spatial dependence of tungiasis cases was estimated using a semi-variogram in ArcGIS Spatial Analyst tool. A lag size of 0.0074196 was used on the semi-variogram to test autocorrelation in tungiasis concentration. The result showed very low spatial autocorrelation which could indicate that tungiasis concentration between two data points in the study area was not significantly affected by the variable distance. Consequently, in future, other geostatical analytical tests like kriging could be tested with more data to establish whether there is directionality in the concentration of tungiasis. Furthermore, it would be interesting to conduct comparative studies in two or more heterogeneous geographical areas to establish if there are environmental factors driving the spread of tungiasis.

### Conclusion

Tungiasis is an important health problem in Vihiga County occasioned by the low economic status of the people affected. Factors that point to poverty contribute to the occurrence of tungiasis in Vihiga County. These findings suggest a need to design control strategies for tungiasis that are cost effective and easily accessible by the communities affected.

## Supporting information

S1 Checklist STROBE checklist. (DOC)

## References

[1] Feldmeier H, Heukelbach J, Ugbomoiko US, Sentongo E, Mbabazi P, Von Samson-Himmelstjerna G, et al Tungiasis—a neglected disease with many challenges for global public health. PLoS neglected tropical diseases. 2014 10 30; 8(10):e3133 10.1371/journal.pntd.0003133 25356978 PMC4214674

[2] Feldmeier H, Eisele M, Sabóia-Moura RC, Heukelbach J: Severe Tungiasis in Underprivileged Communities: Case Series from Brazil. Emerging infectious diseases. 2003 8; 9 (8):949 10.3201/eid0908.030041 12967492 PMC3020603

[3] WHO 2018 lymphatic filariasis epidemiology. http://www.who.int/lymphatic_filariasis/epidemiology/tungiasis/en/.

[4] Njau NN, Wanzala P, Mutugi M, Ariza L, Heukelbach J. Tungiasis (jigger infestation) in rural Kenya, an emerging infectious disease. Retrovirology. 2012 12; 9(1):P37.

[5] Mwangi JN, Ozwara HS, Gicheru MM. Epidemiology of tunga penetrans infestation in selected areas in Kiharu constituency, Murang’a County, Kenya. Tropical diseases, travel medicine and vaccines. 2015 12; 1(1):13. 10.1186/s40794-015-0015-4 PMC5530937 28883944

[6] Waruguru Chiuri S. Prevalence of Tungiasis and its associated risk factors among residents of Kipkelion West Sub-county, Kericho County, Kenya. Doctoral dissertation, Jomo Kenyatta University of Agriculture and Technology, 2016. http://ir.jkuat.ac.ke/bitstream/handle/123456789/2214/Cheuri

[7] Wiese S, Elson L, Reichert F, Mambo B, Feldmeier H. Prevalence, intensity and risk factors of tungiasis in Kilifi County, Kenya: I. Results from a community-based study. PLOS Neglected Tropical Diseases. 2017 10 9; 11(10):e0005925 10.1371/journal.pntd.0005925 28991909 PMC5648262

[8] Wilcke T, Heukelbach J, Moura RC, Kerr-Pontes LR, Feldmeier H. High prevalence of tungiasis in a poor neighbourhood in Fortaleza, Northeast Brazil. Acta Tropica. 2002 9 1; 83(3):255–8. 12204399 10.1016/s0001-706x(02)00133-x

[9] Ugbomoiko US, Ofoezie IE, Heukelbach J. Tungiasis: high prevalence, parasite load, and morbidity in a rural community in Lagos State, Nigeria. International journal of dermatology. 2007 5; 46 (5):475–81. 10.1111/j.1365-4632.2007.03245.x 17472674

[10] Ugbomoiko US, Ariza L, Ofoezie IE, Heukelbach J. Risk factors for tungiasis in Nigeria: identification of targets for effective intervention. PLOS Neglected Tropical Diseases. 2007 12 5; 1(3):e87 10.1371/journal.pntd.0000087 18160986 PMC2154384

[11] Okoth AA. Morbidity, Risk Factors, and flea species responsible for Tungiasis in selected villages in Kisumu County, Kenya. Masters dissertation, Kenyatta University. 2015. http://ir-library.ku.ac.ke/handle/123456789/13298

[12] Heukelbach J. Tungiasis. Revista do Instituto de Medicina Tropical de São Paulo. 2005 12; 47(6):307–13. 16553319 10.1590/s0036-46652005000600001

[13] Muehlen M, Feldmeier H, Wilcke T, Winter B, Heukelbach J. Identifying risk factors for tungiasis and heavy infestation in a resource-poor community in northeast Brazil. Transactions of the Royal Society of Tropical Medicine and Hygiene. 2006 4; 1;100(4):371–80. 10.1016/j.trstmh.2005.06.033 16297946

[14] Girma M, Astatkie A, Asnake S. Prevalence and risk factors of tungiasis among children of Wensho district, southern Ethiopia. BMC infectious diseases. 2018 12; 18(1):456 10.1186/s12879-018-3373-5 30200882 PMC6131746

[15] Wright, K.A. Web GIS as a DIsease Management Workspace: Enabling Advocacy at Multiple Scales Across Multiple Continents with the Case of Tungiasis. Master’s thesis, University of Southern California. 2017. https://spatial.usc.edu/wp-content/uploads/2017/08/Wright_Katherine

[16] Brooker S, Utzinger J. Integrated disease mapping in a polyparasitic world. Geospatial health. 2007; 1(2):141–6. 10.4081/gh.2007.262 18686239

[17] Brooker S, Clements AC, Bundy DA. Global epidemiology, ecology and control of soil-transmitted helminth infections. Advances in parasitology. 2006 1; 62:221–61. 10.1016/S0065-308X(05)62007-6 16647972 PMC1976253

[18] Diggle PJ, Thomson MC, Christensen OF, Rowlingson B, Obsomer V, Gardon J, et al Spatial modelling and the prediction of Loa loa risk: decision making under uncertainty. Annals of Tropical Medicine & Parasitology. 2007 9; 101(6):499–509. 17716433 10.1179/136485913X13789813917463

[19] Brooker S, Rowlands M, Haller L, Savioli L, Bundy DA. Towards an atlas of human helminth infection in sub-Saharan Africa: the use of geographical information systems (GIS). Parasitology Today. 2000 7; 16(7):303–7. 10858650 10.1016/s0169-4758(00)01687-2

[20] Ngugi E. Exploring Kenta’s inequality. Pooling together or pooling apart? 2013. https://www.knbs.or.ke/download/vihiga-county-pdf/

[21] Vihiga County first integrated development plan 2013–2017. https://roggkenya.org/wp-content/uploads/docs/CIDPs/Vihiga-County-Integrated-Development-Plan_CIDP_2013-2017.

[22] Daniel WW (1999). Biostatistics: A Foundation for Analysis in the Health Sciences. 7^th^ edition New York: John Wiley & Sons.

[23] Macfarlane SB. Conducting a descriptive survey: 2. Choosing a sampling strategy. Tropical doctor. 1997 1; 27(1):14–21. 10.1177/004947559702700108 9030013

[24] Naing L, Winn T, Rusli BN. Practical issues in calculating the sample size for prevalence studies. Archives of orofacial Sciences. 2006; 1:9–14.

[25] Eisele M, Heukelbach J, Van Marck E, Mehlhorn H, Meckes O, Franck S, Feldmeier H. Investigations on the biology, epidemiology, pathology and control of Tunga penetrans in Brazil: I. Natural history of tungiasis in man. Parasitology research. 2003 6 1; 90(2):87–99. 12756541 10.1007/s00436-002-0817-y

[26] Tsai PJ, Lin ML, Chu CM, Perng CH. Spatial autocorrelation analysis of health care hotspots in Taiwan in 2006. BMC Public Health. 2009 12; 9 (1):464. 20003460 10.1186/1471-2458-9-464 PMC2799414

[27] Ministry of Health Kenya, division of environmental health. National Policy Guidelines on Prevention and Control of Jigger Infestations; 2014.

[28] Mazigo HD, Bahemana E, Konje ET, Dyegura O, Mnyone LL, Kweka EJ, et al Jigger flea infestation (tungiasis) in rural western Tanzania: high prevalence and severe morbidity. Transactions of the Royal Society of Tropical Medicine and Hygiene. 2012 4; 106(4):259–63. 10.1016/j.trstmh.2011.12.001 22305586

[29] Heukelbach J, Costa AM, Wilcke T, Mencke N, Feldmeier H. The animal reservoir of Tunga penetrans in severely affected communities of north‐east Brazil. Medical and veterinary entomology. 2004 12; 18(4):329–35. 10.1111/j.0269-283X.2004.00532.x 15641998

[30] Ugbomoiko US, Ariza L, Heukelbach J. Pigs are the most important animal reservoir for Tunga penetrans (jigger flea) in rural Nigeria. Tropical Doctor. 2008 10; 38(4):226–7. 10.1258/td.2007.070352 18820191

[31] Pilger D, Schwalfenberg S, Heukelbach J, Witt L, Mehlhorn H, et al Investigations on the biology, epidemiology, pathology, and control of Tunga penetrans in Brazil: VII. The importance of animal reservoirs for human infestation. Parasitology Research. 2008; 102: 875–880. 10.1007/s00436-007-0840-0 18172688

[32] Mutebi F, Krücken J, Feldmeier H, Waiswa C, Mencke N, Sentongo E, et al Animal reservoirs of zoonotic tungiasis in endemic rural villages of Uganda. PLOS Neglected Tropical Diseases. 2015 10; 9(10):e0004126 10.1371/journal.pntd.0004126 26473360 PMC4608570

[33] Unicef-Water, Sanitation and Hygiene. http://www.unicef.org/wash/.

[34] Linardi PM, Calheiros CM, Campelo-Junior EB, Duarte EM, Heukelbach J, Feldmeier H. Occurrence of the off-host life stages of Tunga penetrans (Siphonaptera) in various environments in Brazil. Annals of Tropical Medicine & Parasitology. 2010 6; 104(4):337–45. 20659395 10.1179/136485910X12743554759902

[35] Kimotho S, Miller A, Ngure P. Stigmatizing Beliefs, Stereotypes and Communication Surrounding Tungiasis in Kenya. https://www.researchgate.net/publication/319990259

[36] Wafula ST, Ssemugabo C, Namuhani N, Musoke D, Ssempebwa J, Halage AA. Prevalence and risk factors associated with tungiasis in Mayuge district, Eastern Uganda. The Pan African medical journal. 2016; 24. 10.11604/pamj.2016.24.77.8916 PMC5012786 27642416

[37] Raso G, Vounatsou P, McManus DP, Utzinger J. Bayesian risk maps for Schistosoma mansoni and hookworm mono-infections in a setting where both parasites co-exist. Geospatial health. 2007 11;2(1):85 10.4081/gh.2007.257 18686258 PMC2753301

